# Kinetics and thermodynamics studies of nickel manganite nanoparticle as photocatalyst and fuel additive

**DOI:** 10.1016/j.heliyon.2024.e33861

**Published:** 2024-06-28

**Authors:** Shumaila Fatima, Mahwish Iqbal, Haq Nawaz Bhatti, Norah Alwadai, Maryam Al Huwayz, Arif Nazir, Munawar Iqbal

**Affiliations:** a Environmental Chemistry Laboratory, Department of Chemistry, University of Agriculture Faisalabad, Pakistan; b Department of Physics, College of Science, Princess Nourah bint Abdulrahman University (PNU), Riyadh, 11671, Saudi Arabia; c Department of Chemistry, The University of Lahore, Lahore, Pakistan; d School of Chemistry, University of the Punjab, Lahore 54590, Pakistan

**Keywords:** Photocatalytic degradation, Nickel manganite, Fuel additives, Dye removal, Solar light irradiation

## Abstract

In this study, nickel manganite (NiMn_2_O_4_) nanoparticles were prepared using a hydrothermal method and examined its potential as a photocatalyst for the Acid Green 25 (AG-25) dye degradation. The nanoparticles were subjected to structural analysis using X-ray diffraction (XRD) and morphological analysis using scanning electron microscopy (SEM). The study examined the kinetics and thermodynamics of degradation processes that are catalyzed by photocatalysis. To ascertain their effect on dye degradation, several parameters, such as catalyst dose, H_2_O_2_ concentration, and temperature, were investigated. With a temperature of 315 K in a pseudo-first-order kinetic reaction, a 0.3 M H_2_O_2_ concentration, 0.05 mg/mL catalyst dose, and a promising removal efficiency of 96 % was achieved by the NiMn_2_O_4_ NPs in 40 min. Thermodynamic analysis revealed the spontaneous and entropy-driven nature of catalytic degradation, progressing favorably at elevated temperatures. Additionally, the NiMn_2_O_4_ NPs were applied as a fuel additive to analyze its influence on combustion and the physical characteristics of the modified fuel. The modified fuel demonstrated exceptional catalytic efficiency, emphasizing the potential of the NiMn_2_O_4_ NPs as an effective additive.

## 1 Introduction

Globally, rapid population growth, industrial activities, and urbanization have led to a substantial decline in water resources. In developed nations, the impact of industrialization has resulted in a reported disease, which could escalate further due to the persistent rise in water pollution [[1], [2], [3]]. Numerous routes allow different types of water pollutants, including pesticides, heavy metals, organic dyes, medications, and insecticides, to enter water channels. These include the release of industrial pollutants, the discharge of untreated or insufficiently treated sewage from wastewater treatment plants, and the use of chemicals such as pesticides and fertilizers in agricultural practices [4,5]. Of these pollutants, dyes are particularly prevalent in ecological matrices because of their wide-ranging uses in the printing, dyeing, plastics, mining, pharmaceutical, and textile industries [6]. Due to their stable, complex structures and non-biodegradable nature, dyes present a serious environmental risk. The yearly production of different synthetic dyes surpasses 7 × 10^5^ tons worldwide. About 10,000 tons of dyes are consumed by the textile industry alone each year, which contributes to the annual release of about 100 tons of dyes into waterways. The creatures that live in aquatic ecosystems are negatively impacted by this discharge [7]. Specifically, AG-25 is a 1,4-diamino anthraquinone dye with two terminal sulfonic acid groups. This dye is widely used in the paper, leather, and cosmetic industries as well as for staining silk, wool, and synthetic polyamide. Interestingly, AG-25 is an active component in hair color products found in concentrations as high as 0.3 % [8]. AG-25 is a hazardous water contaminant that can cause burning feelings in the gastrointestinal tract and mouth, skin irritation, and emphysema, among other negative effects. During synthesis and processing, AG-25 is released into water channels, compromising water quality and obstructing light penetration. As a result, this release causes the dissolved oxygen content of the surrounding water and air to decrease [9].

Adsorption, precipitation, coagulation, reverse osmosis, and solvent extraction are just a few of the physicochemical and biological techniques used in conventional wastewater treatment methods [[10], [11], [12], [13]]. Unfortunately, because of their inherent shortcomings, which include difficult handling, lengthy procedures, and the use of expensive chemicals, these established methods frequently prove ineffective in dealing with emerging contaminants. Furthermore, these methods might unintentionally cause toxins to move from one aqueous phase to another, which would add to the production of secondary pollutants [[14], [15], [16]].

Because advanced oxidation techniques are inexpensive, sustainable, and environmentally friendly, they are useful for breaking down dangerous organic contaminants into simpler compounds [17,18]. Semiconducting nanomaterials act as photocatalysts in this process when light energy is present. Photoexcited electrons are promoted from the valence band (filled) to the conduction band (empty) by nano-photocatalysts that absorb photons with energy equal to or greater than their band gap. Radicals are produced as a result of this process, which creates electron-hole pairs. In turn, these radicals help dye molecules break down into less toxic components like carbon dioxide and water [[19], [20], [21], [22]].

Nanoparticles as fuel additives have shown promising effects in enhancing fuel properties, particularly in biodiesel. By incorporating nanoparticles into biodiesel, several benefits can be achieved. Firstly, nanoparticles can improve the ignition characteristics of the fuel, leading to shorter ignition times and more efficient combustion. This can result in increased engine performance, reduced fuel consumption, and lower emissions of pollutants such as carbon monoxide, unburned hydrocarbons, nitrogen oxides, and particulate matter. Additionally, nanoparticles can enhance the stability and viscosity of biodiesel, making it more suitable for use in diesel engines. They can also mitigate issues such as fuel oxidation and degradation, thereby extending the shelf life of the fuel. Moreover, nanoparticles can act as catalysts, promoting more complete combustion and reducing the formation of harmful by-products [23].

The manganese (Mn), nickel (Ni), and their compounds (especially oxides) exhibit promising photocatalytic properties, there are several disadvantages and drawbacks associated with their use as photocatalysts, i.e., Mn and Ni compounds, such as manganese oxide (MnO) and nickel oxide (NiO), typically have narrow band gaps, which may limit their ability to absorb light in the visible spectrum. This can result in relatively low photocatalytic efficiency, especially under solar irradiation, which contains a significant portion of visible light. The quantum efficiency of photocatalytic reactions catalyzed by manganese and nickel compounds may be relatively low, meaning that only a small fraction of absorbed photons leads to the desired chemical transformations. This inefficiency can result in slower reaction rates and the need for longer irradiation times to achieve desired outcomes. Manganese and nickel compounds may suffer from rapid recombination of photogenerated charge carriers (electrons and holes), which reduces the overall efficiency of photocatalytic processes. Effective charge carrier separation and recombination suppression are essential for maximizing the performance of photocatalysts, and strategies to mitigate recombination losses may be necessary [24,25]. On the other hand, NiMn_2_O_4_ possesses several advantageous properties that make it a promising photocatalyst material for various applications, including its efficient visible light absorption, enhanced charge separation, chemical stability, tunable properties, versatility, abundance, cost-effectiveness, and compatibility with composite materials. These advantages position NiMn_2_O_4_ as a promising candidate for addressing critical environmental and energy challenges through photocatalysis [26]. The catalyst (NiMn_2_O_4_) can significantly affect the kinetics of a photocatalytic reaction by providing alternative reaction pathways, lowering the activation energy barrier, and altering the rate of reaction. While the primary role of catalysts in photocatalytic reactions is to accelerate reaction rates by affecting the kinetics of the process, they can also indirectly influence the thermodynamics of the reaction by providing alternative reaction pathways, modifying energy levels, and altering the equilibrium position. Understanding the interplay between kinetics and thermodynamics is crucial for designing efficient photocatalytic systems and optimizing reaction conditions for desired outcomes [27].

Many metallic photocatalysts have been used to remove harmful contaminants from water, but NiMn_2_O_4_ has not yet been investigated as a possible solution. Previously, *Vernonia amygdalina* leaf extract was used to synthesize MnO_2_/NiO nanoparticles, which were then used as an anode modifier catalyst in microbial fuel cells (MFCs) [28]. Furthermore, NiMn_2_O_4_ nanoparticles were employed as supercapacitor electrode materials; these were produced using hydrothermal techniques aided by microwaves [29]. Moreover, a colloidal solution method-prepared NiMn_2_O_4_/rGO composite demonstrated efficacy in electrochemical applications [30]. Consequently, it is essential to research the photocatalytic efficiency of NiMn_2_O_4_ when exposed to sunlight, as this is a sustainable and green method. Combining metals improves the characteristics of each metal separately and modifies the electronic surface properties. NiMn_2_O_4_ is particularly useful as a cathode material in lithium-ion batteries and as a magnetic component in permanent magnets, electroplating, and electrodeless plating processes due to its high electrical conductivity, thermal stability, and magnetic susceptibility [31,32]. Many physicochemical techniques are used in the synthesis of nanomaterials, the hydrothermal synthesis method offers several advantages, including controlled morphology, homogeneous particle distribution, high purity & phase homogeneity, scalability, and energy efficiency when performed at low temperature and versatility, making it a preferred choice for preparing nanoparticles for various applications [33].

One of the main causes of pollution in the environment is still the release of harmful gases during fuel combustion. Tight emission regulations have encouraged many researchers to investigate new approaches to solving this problem. To reduce pollution, exhaust gases must be treated before being released into the environment. One novel approach to lowering harmful gas emissions and improving fuel efficiency is the incorporation of nanoscale materials into fuel [34]. Because of their high water-holding capacity, resistance to corrosion, and chemical and thermal stability, nickel manganite nanoparticles are an excellent choice for fuel additives.

The current work focuses on the hydrothermal synthesis of rod-shaped bimetallic nickel manganite NPs. The AG-25 dye was degraded using the synthesized NP material, and different parameters like temperature, H_2_O_2_ concentration, and catalyst dose were optimized. To further increase the adaptability of the created material, NiMn_2_O_4_ NPs were added to the fuel to study the combustion and physical characteristics of the modified fuel.

## 2 Materials and methods

The following materials were used in their original forms: cetyltrimethylammonium bromide (CTAB; 99 %, ITW Reagents), ethanol (99.8 %, Sigma-Aldrich), manganese (II) nitrate tetrahydrate (98 %, Sigma-Aldrich), nickel (II) nitrate hexahydrate (≥97.0 %, Sigma-Aldrich), and aqueous ammonia (25 %, Emsure). The supplier of AG-25 dye (C_28_H_20_N_2_Na_2_O_8_S_2_) was Sigma-Aldrich. Distilled water was used to prepare all of the solutions used in this investigation.

### 2.1 Synthesis of nickel manganite NPs

To prepare NiMn_2_O_4_ NPs, a hydrothermal method was used. First, 40 mL of a water and ethanol (2:1) mixture was used to dissolve the necessary amount of salt, resulting in a 0.15 M solution of Nickel (II) nitrate hexahydrate. Concurrently, a distinct solution of Manganese (II) nitrate tetrahydrate (0.2 M) was made in 40 mL of a 2:1 water: ethanol mixture. Both solutions were mixed while being constantly stirred. The solution was then stirred for 20 min after the addition of 1 g of cetyltrimethylammonium bromide (CTAB). After that, vigorous stirring was used to gradually add aqueous ammonia until the pH of the mixture reached the range of 10–12. After that, the mixture was put in an electric oven and kept at 110 °C for 6 h in a stainless-steel autoclave lined with Teflon. Centrifugation at 4000 rpm was used to separate the synthesized sample from the reaction mixture, and it was then cleaned with ethanol and distilled water (DW). After that, the final product was oven-dried at 60 °C and then further calcined for 4 h at 500 °C.

### 2.2 Characterization

Using an SEM (JEOL JMT 300), the morphological characteristics of the synthesized materials were examined. X-ray diffractometer (P'analytical X′ Pert PRO) was used for structural analysis. A sample of commercial diesel was obtained from Pakistan State Oil (PSO), located in Pakistan. With the ASTM D93 working standard, a flash point tester (APEX-JCX309) was used to measure the fire and flash points. With the ASTM D1298 operating mode, a Gravity meter (DA-640) was used to measure the specific gravity of diesel. Using the ASTM D240 working standard, an oxygen bomb calorimeter (APEX-JCX406) was used to measure the fuel calorific values. A digital viscometer was used to measure kinematic viscosity by ASTM D445 guidelines. We used a digital thermometer to measure the cloud and pour points. At *λ*_max_ 640 nm, a UV spectrophotometer (Cecil CE7200 7000-series) was utilized to analyze the residual dye concentration following each trial.

### 2.3 Photocatalytic activity

The degradation of Acid Green 25 dye under solar radiation was used to gauge the synthesized sample's catalytic efficacy. A 40 mg/L dye solution contained 0.01 g of catalyst that was distributed for every trial. The mixtures were first exposed to sunlight irradiation after being continuously stirred in the dark for 30 min to establish adsorption-desorption equilibrium. Numerous process variables were examined, such as temperature fluctuations (between 300 and 315 K), catalyst dose (between 0.01 and 0.05 mg/mL), and H_2_O_2_ concentration (between 0.1 and 0.3 M). Measurements of absorbance at *λ*_max_ (640 nm) were made after 3 mL of the reaction mixture was taken out at different times to track the remaining concentration of AG 25 dye. Using an oil bath, the temperatures necessary for thermal catalytic degradation were found. The catalytic efficacy of NiMn_2_O_4_ for AG-25 was analyzed through Beer's law using equation (1).

(1) $$D\left(\%\right)=\frac{C_{o}-C_{t}}{C_{o}}\times 100$$

where, C_o_ and C_t_ indicate the absorbance at zero time and absorbance at a time ‘t', respectively.

#### 2.3.1 Kinetics study

The Langmuir–Hinshelwood kinetics model is a widely used mathematical framework in heterogeneous catalysis, particularly in surface reactions, which describes the rate of a chemical reaction that occurs on the surface of a solid catalyst. In this model, the reactant molecules adsorb onto the surface of the catalyst, forming temporary bonds or intermediates. These adsorbed species then react with each other to form products, which subsequently desorb from the catalyst surface. The rate of reaction is determined by the kinetics of adsorption, desorption, and surface reaction steps [35]. In the presence of sunlight and a hydrogen peroxide promoter, the NiMn_2_O_4_ NPs promoted the photocatalytic degradation of Acid Green 25. Using a UV–visible spectrophotometer, the wavelength of 640 nm was specifically selected to measure the residual pollutant concentration at various time intervals. The time-dependent wavelength scan UV–visible spectrum of AG-25 shows a notable decrease in absorbance at 640 nm. This observation shows that the dye is degrading, highlighting the NiMn_2_O_4_ NP efficiency as a nanocatalyst when exposed to sunlight. The oxidant concentration is kept larger than the dye so that photocatalysis follows pseudo-first-order kinetics as stated by equation (2).

(2) $$\ln\left(\frac{A_{t}}{A_{o}}\right)=-k_{aap}\times t$$

where k_aap_ represents the apparent rate constant and A_o_ is absorbance at t = 0 and A_t_ indicates absorbance at t = t.

#### 2.3.2 Thermodynamic study

Eyring and Arrhenius's equations were used to further analyze the data from the thermal catalytic degradation of AG-25 by NiMn_2_O_4_ NPs to conduct a thermodynamic study of this reaction. Equation (3) explains the effect of temperature on the apparent rate constant (k_aap_) using the Arrhenius equation.

(3) $${lnk}_{app\;}=lnA-\frac{E_{a}}{RT}$$

The parameters of 1/T and lnk_aap_ have an inverse relationship, as established by the Arrhenius equation. 1/T declines with increasing temperature, and as a result, lnk_aap_ shows an increase in the Arrhenius plot. This suggests that at higher temperatures, the rate of degradation was enhanced. As a result, pseudo-first-order kinetics is demonstrated by the Arrhenius plot, which shows a linear relationship between temperature and lnk_aap_. As mentioned in Eq. (4), the Eyring equation is used to further explore the relationship between the apparent rate constant and temperature.

(4) $$\ln\frac{k_{app}}{T}=\ln\frac{K_{b}}{h}+\frac{{\Delta S}^{*}}{R}-\frac{{\Delta H}^{*}}{RT}$$

According to the Eyring plot, as the temperature rises, the value of lnk_aap_/T rises as well, suggesting that the degradation rate also increases at higher temperatures. The degradation of AG-25 appears to follow the Eyring equation, based on the degradation rate's linear increase with temperature. Using Arrhenius and Eyring plots, thermodynamic parameters for the dye's thermal catalytic degradation are also calculated.

### 2.4 Applications as fuel additives

By using these particles in varying concentrations, the impact of NiMn_2_O_4_ NPs on the fuel's physical characteristics and combustion was assessed. A variety of parameters were examined with and without the addition of NiMn_2_O_4_ NPs, including the test fuel's calorific values, cloud and pour points, specific gravity, kinematic viscosity, and flash and fire points.

## 3 Results and discussion

### 3.1 Properties of the NiMn_2_O_4_ NPs

**Fig. 1:**
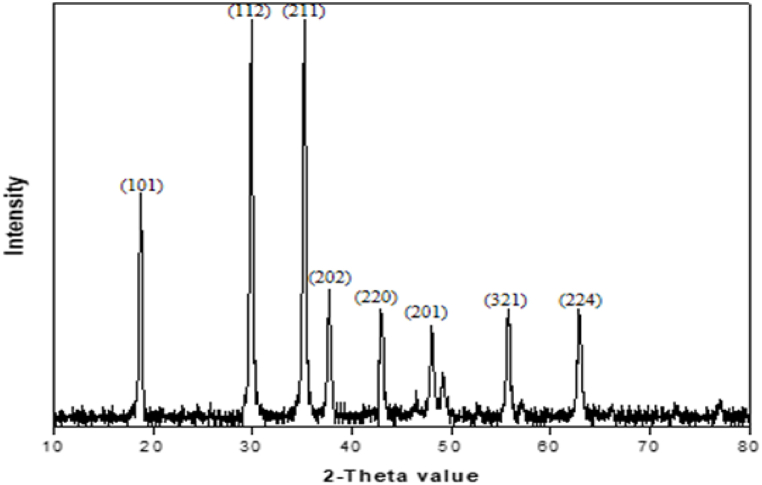
XRD pattern of the prepared NiMn_2_O_4_ heterostructure.

X-ray diffraction analysis was used to determine the phases and crystalline structure of NiMn_2_O_4_. The NiMn_2_O_4_ NPs XRD pattern, as shown in Fig. 1, has distinct peaks at 2-Theta values, which correspond to the Miller planes of (111), (220), (311), (242), (400), (331), (511) and (440). The results validate the NiMn_2_O_4_ NP cubic crystal structure formation. The JCPDS card number 01-071-0852 was in agreement with the observed diffraction peaks [35]. The XRD pattern of NiMn_2_O_4_ exhibits sharp and intense peaks, which suggest the absence of any impurities in the synthesized product. The particle size was estimated using the Scherrer equation, which was in 9.04–69.5 nm with an average particle size of 29.54 nm.

**Fig. 2:**
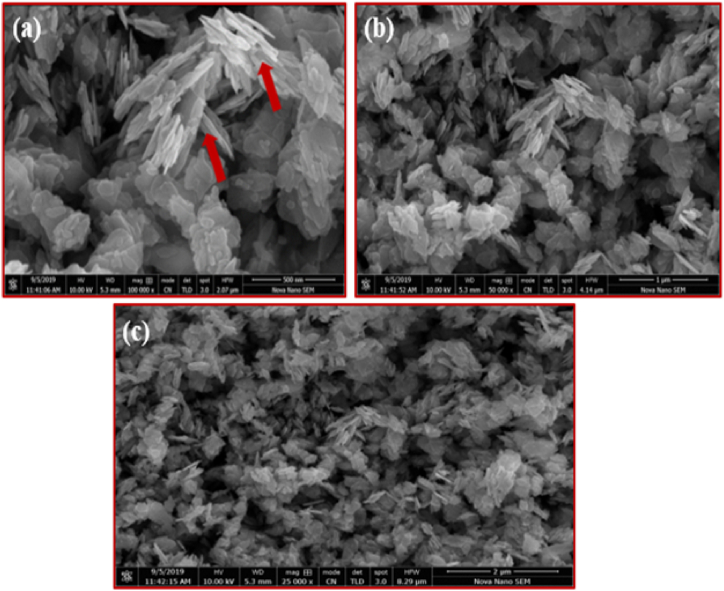
Surface morphology of the prepared NiMn_2_O_4_ heterostructure, (a) 500 nm, (b) 1 μm and (c) 2 μm.

SEM analysis was used to characterize the morphology of the NiMn_2_O_4_ NPs [36]. The produced images, which are shown in Fig. 2a, show that the synthesized product has a rod- and needle-shaped morphology. Interestingly, at some locations, the particles show interconnectivity and some of them have a spherical shape that contributes to an oval morphology. There is proof of particle stacking in Fig. 2b. Due to the irregular shapes of the particles, the product exhibits polydispersity. An uneven arrangement of particles that are oriented both parallel and antiparallel to one another is shown in Fig. 2c. The distinct edges of the particles show minimal agglomeration despite this arrangement. In addition, it appears that at certain locations, microscopic broken particles are adhered to the surface [37].

### 3.2 Point of zero charges (pH_PZC_) analysis

To analyze the pH_PZC_ of NiMn_2_O_4_, a 100 mL NaCl aqueous solution series (0.1 M) with initial pH values adjusted between 3 and 11 was prepared. For each catalyst, about 0.01 g of NiMn_2_O_4_ was added to each solution and after 24 h, the final pH was recorded. To find the catalyst's pH_pzc_, the data was plotted between ΔpH and pH values. The pHpzc is considered where the curve passes through the zero point at ΔpH axis and the NiMn_2_O_4_ pHpzc value was found to be 7.12. A pH_PZC_ value of 7.12 indicates that the catalyst surface is electrically neutral. Below this pH value, the surface of the catalyst will carry a net positive charge due to the protonation of surface functional groups, while above this pH value, the surface will carry a net negative charge due to the deprotonation of surface functional groups. The pH_PZC_ is an important parameter in understanding the surface chemistry and reactivity of a catalyst, particularly in catalytic processes involving aqueous solutions. It influences the adsorption of reactant dye molecules and the interaction between the catalyst surface and the surrounding environment. Since AG25 is an anionic dye, it carries a negative charge in solution. At a pH below the pH_PZC_ of NiMn_2_O_4_ (7.12), the catalyst surface would be positively charged. This electrostatic attraction between the positively charged catalyst surface and the negatively charged dye molecules could enhance the adsorption of the dye onto the catalyst surface, facilitating the degradation process [38].

### 3.3 Photocatalytic performance

#### 3.3.1 Effect of catalyst dose

**Fig. 3:**
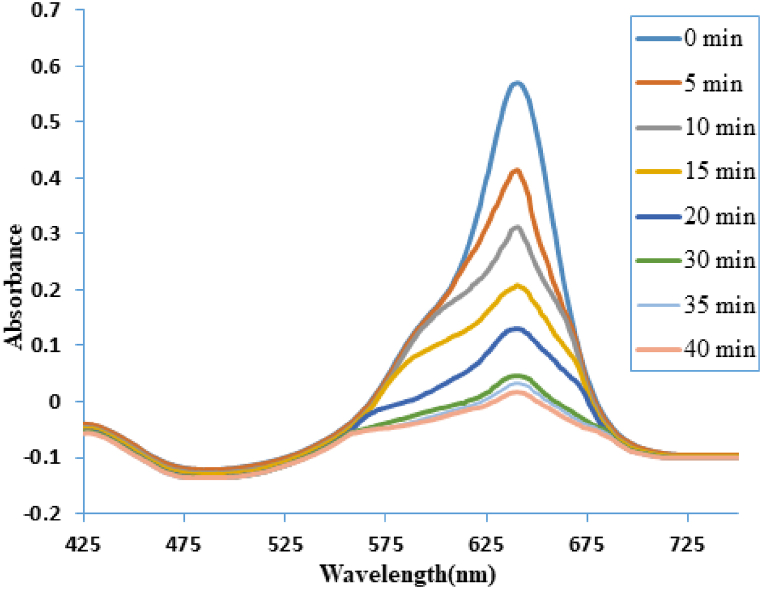
UV–visible spectra of the AG-25 treated by NiMn_2_O_4_ heterostructure at various irradiation durations.

**Fig. 4:**
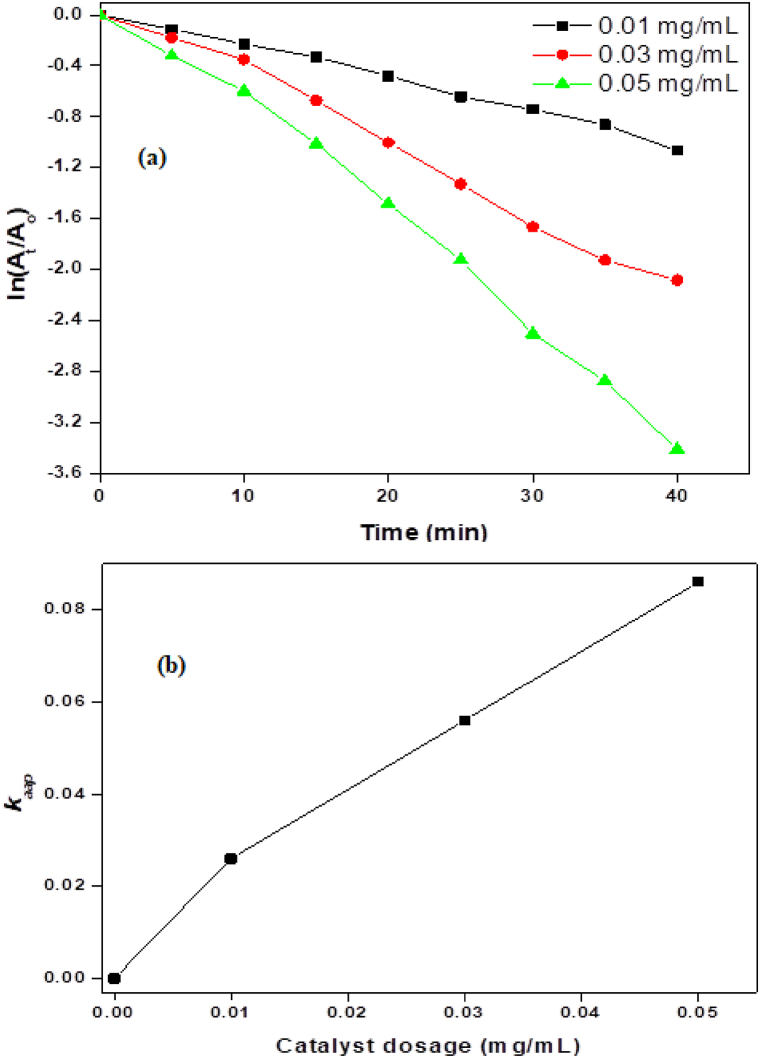
(a) Influence of catalyst dose on AG-25 degradation and (b) rate constant versus dose of catalyst.

The amount of catalyst used has a complicated effect on dye degradation that is dependent on several variables. Although higher catalyst doses generally result in better degradation efficiency, using too high of a dose may have practical drawbacks and diminishing returns. Several factors need to be taken into account to optimize the catalyst dose, including mass transport effects, catalyst properties, reaction kinetics, and economic considerations. It usually takes experimental research to find the ideal catalyst dose for a given dye degradation process [39]. To maximize the photocatalytic activity of the produced product, the effect of catalyst dosage on the rate of photocatalytic degradation using bimetallic NiMn_2_O_4_ NPs was examined at different doses (0.01, 0.03, and 0.05 mg/mL) under solar irradiation and UV–visible spectra of dye absorbance versus time is depicted in Fig. 3. The results were plotted as ln(A_t_/A_o_) versus time using a dye solution at a constant concentration of 40 ppm (Fig. 4a). The ln(A_t_/A_o_) plot shows a notable decrease after a slower initial decline, referred to as the induction period, and an increase in the degradation rate. As the reaction continues, eventually the rate of degradation becomes constant. According to pseudo-first-order kinetics, the decrease in the ln(A_t_/A_o_) value with time showed a linear trend, suggesting that NiMn_2_O_4_ has excellent photocatalytic capabilities for dye degradation. When NiMn_2_O_4_ NPs are present, AG-25 degrades and exhibits a 5-min retention period at catalyst dosages of 0.01 mg/mL and 0.03 mg/mL. The retention period, however, approaches zero minutes at 0.05 mg/mL, suggesting that while the rate of dye degradation increases with an increase in catalyst dosage, it does not affect the reaction's overall progress time. As the dose of NiMn_2_O_4_ particles is increased, the values of k_aap_ likewise rise, suggesting that the degradation rate increases as the catalyst dosage is increased (Fig. 4b, Table 1). This is explained by the fact that there are more active sites on the surface of the NiMn_2_O_4_ catalyst, which permits more dye molecules to adsorb and degrade further. Previous studies [35,36] have reported a similar trend in dye degradation using different catalysts.

**Table 1:** Photocatalytic removal of AG-25 at various conditions (the standard deviation values is 1.9–2.8 % in the *k*_*aap*_ values).

Factors	Conditions	*k*_*aap*_	Retention period (t_r_) (min)	Total progress time (t_p_) (min)	R^2^
Catalyst dose (mg/mL)	0.01	0.026	5	40	0.994
	0.03	0.056	5	40	0.991
	0.05	0.086	0	40	0.992
Oxidant concentration (M)	0.1	0.028	10	40	0.993
	0.2	0.054	5	40	0.994
	0.3	0.086	0	40	0.992
Temperature (K)	300	0.024	10	40	0.994
	305	0.045	5	40	0.993
	310	0.072	0	35	0.996
	315	0.098	0	35	0.992

#### 3.3.2 Effect of oxidant (H_2_O_2_) concentration

**Fig. 5:**
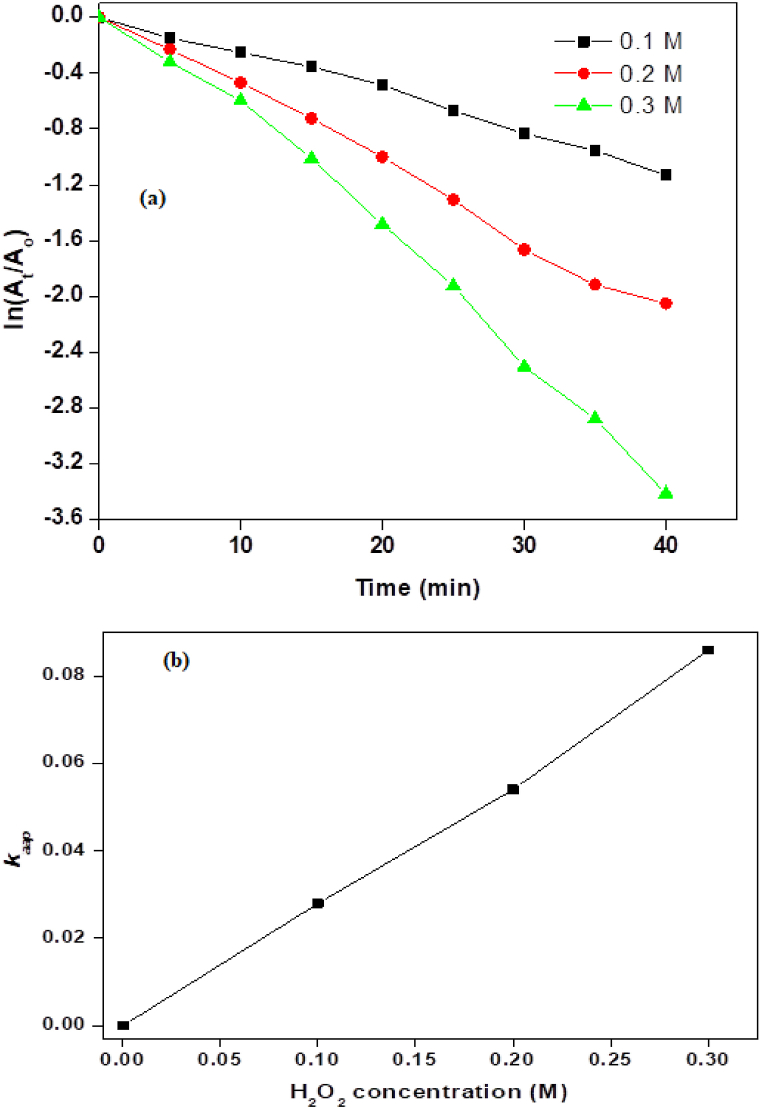
(a) Influence of H_2_O_2_ concentration on AG-25 degradation and (b) rate constant versus H_2_O_2_ concentration.

Hydrogen peroxide can act as a source of reactive oxygen species (ROS) when activated by the photocatalyst under irradiation. These ROS, such as hydroxyl radicals (^•^OH) and superoxide radicals (^•^O_2_^−^), are highly reactive and can effectively oxidize organic dye molecules, leading to enhanced degradation. The synergy may arise from the simultaneous generation of ROS by the photocatalyst and hydrogen peroxide, leading to enhanced oxidation of dye molecules. Hydrogen peroxide solutions typically have acidic or slightly acidic pH values, which can help buffer the pH of the reaction mixture during photocatalytic dye degradation [39]. To improve the material's photocatalytic efficiency for AG-25 dye degradation, hydrogen peroxide (H_2_O_2_) was used as an oxidizing agent. Using an optimal amount of NiMn_2_O_4_, the effects of hydrogen peroxide were examined at different concentrations (0.1, 0.2, and 0.3 M). The ln (A_t_/A_o_) value continuously drops as the concentration of the oxidant in the reaction mixture rises, as shown in Fig. 5a. The reduction is most noticeable at 0.3 M oxidant concentration. The results of the analysis show that using higher oxidant concentrations shortens the retention period. A retention period of 10 min is seen at 0.1 M concentration of hydrogen peroxide, which drops to 5 min at 0.2 M concentration, and approaches zero minutes when the concentration is increased from 0.2 M to 0.3 M. The k_aap_ value rises with increasing oxidant concentration, suggesting a faster rate of dye degradation (Fig. 5b. Table 1). The availability of more oxygenated species is thought to be the cause of this increase in degradation rate, as it speeds up dye degradation when exposed to sunlight [40]. It's interesting to note that the total reaction progress time, which stays constant at 40 min for all concentrations, was not significantly impacted by the increased oxidant concentration.

### 3.4 Effect of temperature

**Fig. 6:**
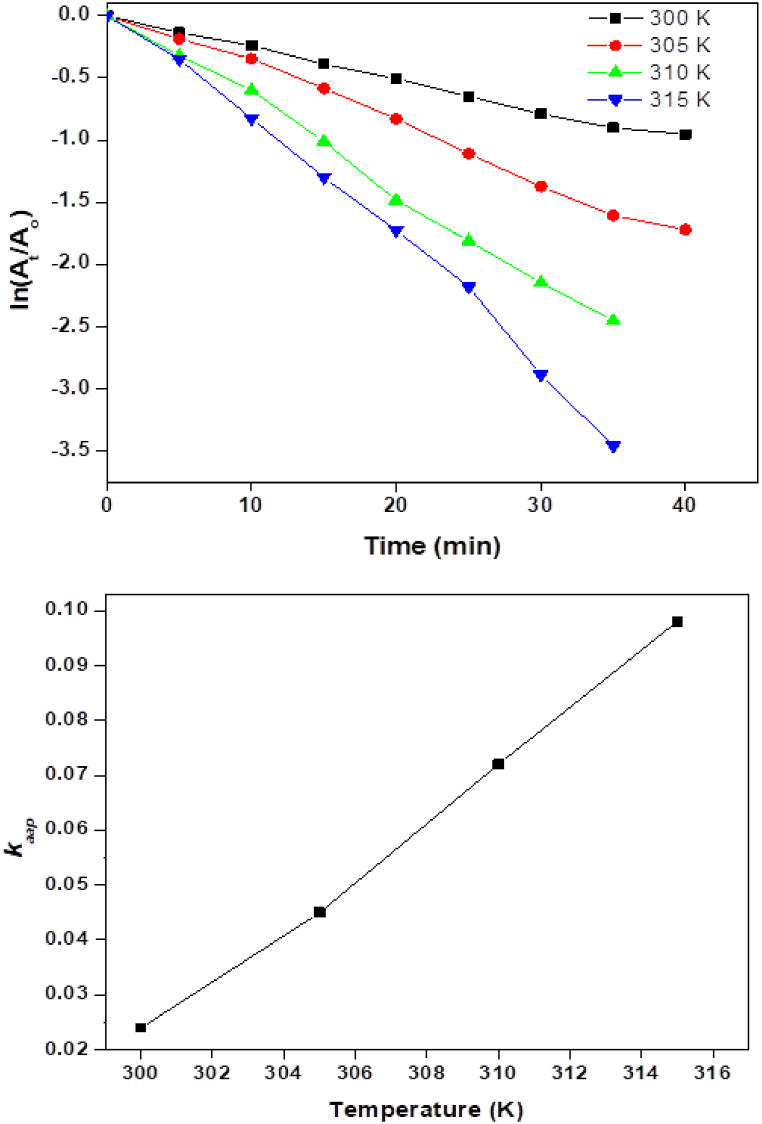
(a) Influence of temperature on AG-25 degradation and (b) rate constant versus temperature.

The effect of temperature on photocatalytic dye degradation can significantly influence the efficiency and kinetics of the degradation process. For endotherm reactions, increasing the temperature enhances the rate of photocatalytic dye degradation. Higher temperatures provide more thermal energy to the system, which can accelerate the chemical reactions involved in the degradation process. Photocatalytic reactions typically have an activation energy barrier that must be overcome for the reaction to proceed. Increasing the temperature decreases this activation energy, making it easier for the photocatalyst to initiate and sustain the degradation reactions. Temperature affects the kinetics of surface reactions occurring on the photocatalyst surface. At elevated temperatures, the surface reaction rates increase due to enhanced molecular mobility and reaction kinetics [41]. While keeping the ideal catalyst dose and H_2_O_2_ concentration, the effects of temperature on the degradation of AG dye catalyzed by bimetallic NiMn_2_O_4_ were examined in the range of 300–315 K. To reach the proper temperatures, the catalyst and H_2_O_2_-loaded dye solution were heated in an oil bath while being stirred. Fig. 6a plots the results of AG-25's thermal catalytic degradation at different temperatures as ln (A_t_/A_o_) versus time. The ln (A_t_/A_o_) plot shows a gradual decrease at 300 K, and the decrease continues to be slow as the temperature is raised gradually from 300 K to 305 K. However, the absorbance decreases more noticeably as the temperature rises above 305 K, especially when it reaches 310 K and 315 K. This linear decline in ln (At/Ao) values suggests pseudo-first-order kinetics for the degradation reaction. The retention periods for the catalytic thermal degradation of AG-25 are 5 min at 305 K and 10 min at 300 K. The retention period drops to 0 min as the temperature rises from 305 K to 315 K. As the temperature rises from 305 K to 310 K and 315 K, the catalytic thermal degradation of AG-25 takes 35 min to complete instead of 40. The apparent rate constant (k_aap_) value increases significantly with each temperature increase from 300 K to 315 K, as shown in Fig. 6b and Table 1. This increase is explained by the system's increased kinetic energy, which raises the collision number and, as a result, increases the number of dye molecules involved in the degradation reaction [[42], [43], [44]].

**Fig. 7:**
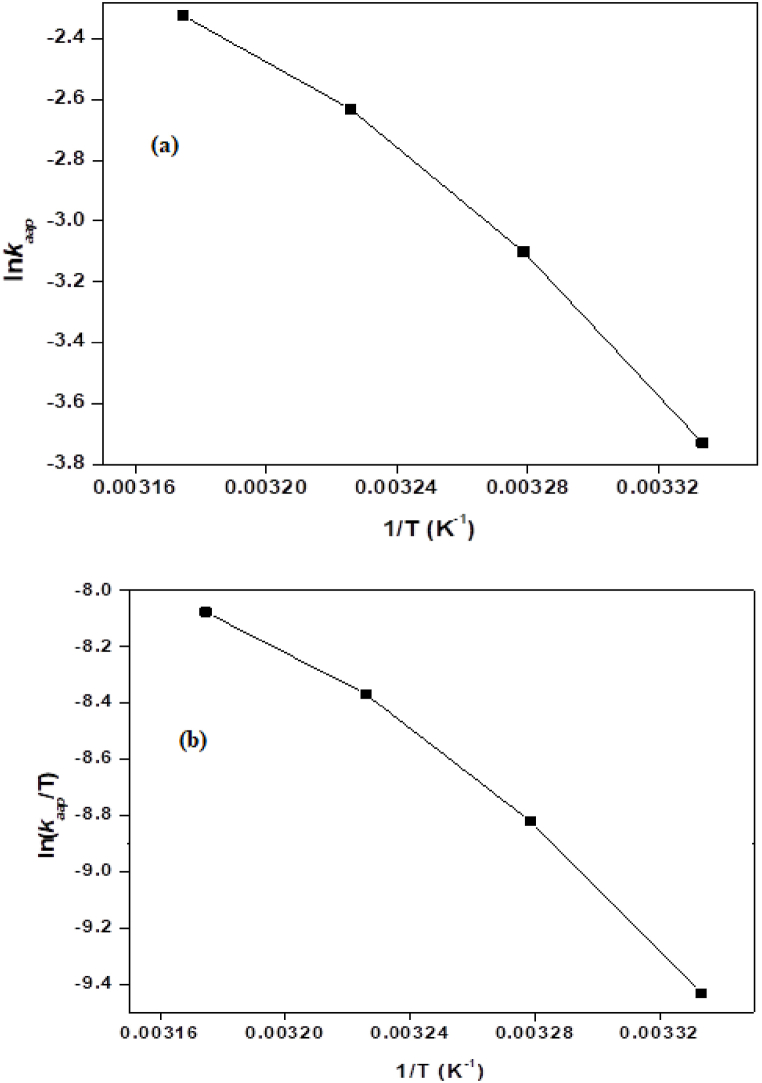
Kinetics plots, (a) Arrhenius plot and (b) Eyring plot.

The parameters "ΔH" (enthalpy of activation) and "ΔS" (entropy of activation) are derived from the Eyring plot using the Eyring equation and are shown in Table 2. The Arrhenius plot (Fig. 7a) is utilized to compute the parameters “Ea” (energy of activation) and A' (collision frequency). The Eyring plot is depicted in Fig. 7b and it was observed that at high temperatures, the catalytic degradation of AG-25 is endothermic and advances, as indicated by the positive ΔH* value, which is greater than zero. The reaction appears to be spontaneous and thermodynamically favorable based on the positive ΔS value. As a result, this reaction advances at high temperatures, quickly converting dye molecules into other products.

**Table 2:** Thermodynamic factors are determined through Arrhenius and Eyring equations.

Catalyst	Arrhenius equation		Eyring equation	
	*E*_*a*_ (Jmol^−1^)	*A*(S^−1^)	${\Delta H}^{*}$ (Jmol^−1^)	${\Delta S}^{*}$ (Jmol^−1^)
NiMn_2_O_4_ NPs	7 × 10^4^	8.91 × 10^25^	7 × 10^4^	159.79

### 3.5 Dye degradation mechanism

**Fig. 8:**
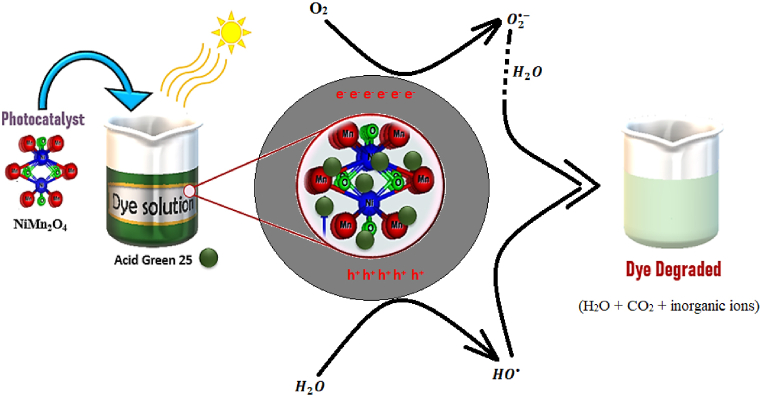
Dye degradation mechanism using catalyst under irradiation.

Fig. 8 shows the steps involved in the degradation mechanism of AG25 dye under irradiation using the NiMn_2_O_4_ catalyst, along with the corresponding chemical reactions (Eqs. (5), (6), (7), (8), (9), (10))). When exposed to light, this advanced oxidation process takes place in several phases, assisted by nanoparticles. The catalyst produces electron-hole pairs (e^−^, h^+^) when exposed to light [39]. At the surface of nanoparticles, electrons (e^−^) interact with oxygen and water molecules to produce highly reactive oxygen species, such as superoxide radicals. In the meantime, hydroxyl radicals are created when water molecules interact with the holes (h^+^). Electrostatic interactions cause the dye molecules in the solution to stick to the surface of the nanoparticle. Reactive oxygen species that are produced interact with the dye molecules that have been adsorbed, starting the degradation process. Notably, by rupturing chemical bonds within dye molecules, hydroxyl radicals significantly contribute to the oxidation of organic compounds. This breakdown proceeds through a series of oxidation stages, which eventually cause the dye to mineralize into smaller, less toxic byproducts like water, carbon dioxide, and inorganic ions. The enhanced efficiency of this advanced oxidation process stems from the high surface area and catalytic activity of nanoparticles, which provide ample sites for reactive oxygen species generation and facilitate dye molecule adsorption. Moreover, the semiconductor properties of nanoparticles enable electron and hole transfer, crucial for reactive oxygen species formation and dye degradation [45].

(5) $${\text{Ni}{\text{Mn}}_{2}O}_{4}+h\upsilon \to \;e^{-}\left(\text{CB}\right)+h^{+}\left(\text{VB}\right)$$

(6) $$O_{2}+\left(e^{-}\right)\to \;O_{2}^{\cdot -}$$

(7) $$H_{2}O+\left(h^{+}\right)\;\to \;{\text{OH}}^{\cdot }$$

(8) $$H_{2}O+O_{2}^{\cdot -}\to \;{\text{OOH}}^{\cdot }+{\text{OH}}^{-}\;\to \;H_{2}O_{2}$$

(9) $$H_{2}O_{2}+O_{2}^{\cdot -}\to \;{\text{OH}}^{\cdot }+{\text{OH}}^{-}$$

(10) $$\text{AG}25\;\text{dye}+{\text{OH}}^{\cdot }+O_{2}^{\cdot -}\to \;H_{2}O+{\text{CO}}_{2}+\text{inorganic}\;\text{ions}$$

### 3.6 Recycling of catalyst

**Fig. 9:**
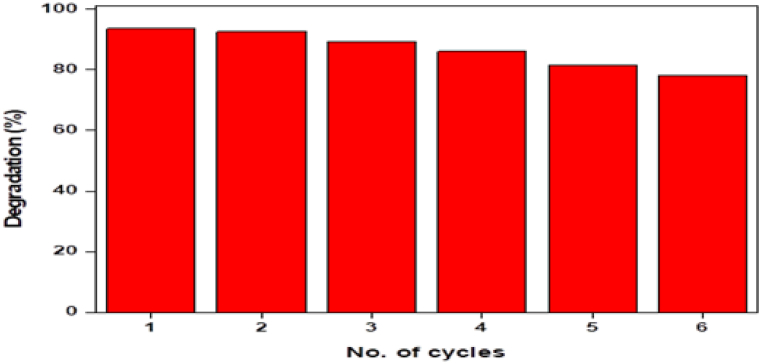
Recycling and reusability of NiMn_2_O_4_ heterostructure.

For the catalyst to be used in a variety of fields, it must be recyclable. To evaluate the catalytic activity of NiMn_2_O_4_, six cycles of degradation experiments were carried out. The nanocatalysts were centrifuged and cleaned with distilled water following each degradation experiment as part of the recycling study before being used again. Fig. 9 shows the outcomes at the end of each cycle. With only an 88.5 % decrease in removal efficiency after three cycles and a 77 % decrease after six cycles, NiMn_2_O_4_ NPs showed good reusability. NiMn_2_O_4_ NPs are an example of a recyclable catalyst that provides reduced waste generation, sustainability, and economic efficiency. Reusable catalysts minimize environmental impact, are environmentally friendly, and contribute to cost-effectiveness. They reduce reliance on raw materials, offer stable processes, and deliver consistent performance. Recycling lessens the production and disposal footprint and increases overall environmental sustainability by extending the catalyst's lifespan. Catalyst recyclability is advantageous in a variety of applications and industries due to this multifaceted benefit.

### 3.7 NiMn_2_O_4_ as the fuel additives

To further explore the potential uses of the synthesized bimetallic NiMn_2_O_4_ NPs, their potential as a fuel additive material was examined to evaluate their effects on the fuel's physical and combustion properties. As the test fuel, diesel was used, and different concentrations of NiMn_2_O_4_ were added to examine various fuel parameters.

**Fig. 10:**
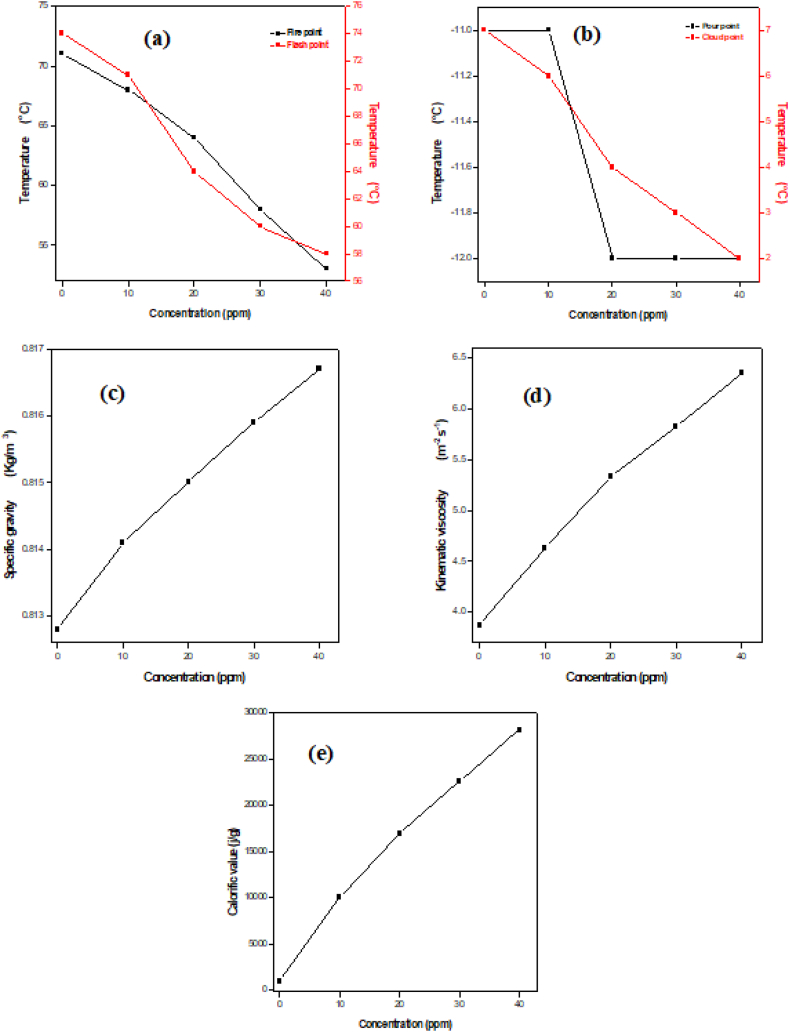
NiMn_2_O_4_ addition impact on fuel properties, (a) flash and fire points, (b) pour and cloud and points, (c) specific gravity, (d) kinematic viscosity and (e) calorific values.

Following ASTM D93 guidelines, the “flash and fire” point temperatures of pure diesel and diesel loaded with NiMn_2_O_4_ NPs at various concentrations (0, 10, 20, 30, & 40 ppm) were observed. The findings, which are shown in Fig. 10a, show that adding NiMn_2_O_4_ NPs to diesel consistently lowers the temperature at both the flash and fire points. The significant decrease in NiMn_2_O_4_ particle concentration is ascribed to the catalyst's substantial specific surface area. As such, the concentration of nanoparticles affects the flash and fire point temperatures of diesel. By catalyzing fuel ignition at faster rates, NiMn_2_O_4_ NPs help to lower the amount of exhaust gas emissions released into the atmosphere. Additionally, these nanoparticles supply oxygen, which helps to convert CO in the exhaust to CO_2_.

Using a digital thermometer in compliance with ASTM D2500 standards, the “pour and cloud point” temperatures of the pure fuel and the fuel mixed with NiMn_2_O_4_ NPs at different concentrations (0, 10, 20, 30, & 40 ppm) were determined. The findings, which are shown in Fig. 10b, show that the diesel with NiMn_2_O_4_ NPs added had a lower cloud point than the diesel without additive particles. Moreover, the cloud point drops noticeably as NiMn_2_O_4_ NP concentration rises, indicating a noteworthy effect on the physical characteristics of diesel. This suggests that these particles can function as material additives with effectiveness. Nevertheless, the addition of the additive material does not significantly alter the fuel's pour point.

By ASTM D1298 guidelines, the “specific gravity” of the supplied fuel at various NiMn2O4 concentrations was determined using a gravity meter. The specific gravity values obtained at different concentrations of NiMn_2_O_4_ NPs (0, 10, 20, 30, & 40 ppm) are displayed in Fig. 10c. Diesel's specific gravity rises when NiMn_2_O_4_ NPs are added, indicating that the fuel's physical characteristics are catalyzed due to the nanoparticles' small size. As a result, the energy content of the fuel increases along with specific gravity, increasing engine efficiency.

Using a viscometer and ASTM D445 standards, the “kinematic viscosity” of diesel at different concentrations of NiMn_2_O_4_ (0, 10, 20, 30, & 40 ppm) was measured. Fig. 10d shows the measured fuel viscosity at various additive concentrations. The addition of NiMn_2_O_4_ NPs is observed to cause an upward trend in the fuel's kinematic viscosity. These particles' small size and large specific surface area, which cause them to attach in layers within the fuel, are responsible for the increase in kinematic viscosity. As a result, the engine runs more efficiently because the fuel flow is less turbulent.

Using an oxygen bomb calorimeter and adhering to ASTM D240 guidelines, the “calorific values” of commercial diesel were determined both with and without NiMn_2_O_4_ NPs at different concentrations. The findings are shown in Fig. 10e, which shows that the fuel loaded with NiMn_2_O_4_ nanoparticles has a higher calorific value than the fuel without these additions. Furthermore, the fuel's calorific values show a linear increase with increasing NiMn_2_O_4_ concentration. The fuel's oxygenated active sites are more readily available when NiMn_2_O_4_ NPs are present, which increases the number of fuel molecules that can adsorb on these sites. In addition to accelerating combustion, this lowers the number of pollutants that are released into the atmosphere.

The nanoparticles as fuel additives can significantly impact various fuel properties, offering several potential benefits, i.e., combustion Efficiency: Nanoparticles, such as metal oxides or carbon-based materials, can enhance combustion efficiency by promoting more complete fuel oxidation. Their high surface area facilitates better fuel-air mixing, improving combustion kinetics and reducing emissions. Certain nanoparticles can modify the ignition characteristics of fuel, either by lowering the ignition temperature or by promoting more uniform ignition across the fuel mixture. Nanoparticles can stabilize fuel formulations by preventing sedimentation or phase separation, thus improving fuel blends' stability and shelf life [23]. This is particularly relevant for biofuels or ethanol or biodiesel fuel blends [46]. By promoting more complete combustion, nanoparticles can help reduce emissions of harmful pollutants such as particulate matter (PM), nitrogen oxides (NOx), and unburned hydrocarbons (HC). Additionally, certain nanoparticles may facilitate the conversion of exhaust gases into less harmful species through catalytic processes. Nanoparticles with lubricating properties, such as graphene or certain metal oxides, can reduce friction and wear within the engine components, leading to improved engine longevity and efficiency. Nanoparticles dispersed in fuel can alter heat transfer characteristics within the engine, potentially leading to more efficient heat dissipation and reduced thermal stress on engine components. Improvements in combustion efficiency, reduced friction, and optimized engine operation can collectively contribute to better fuel economy, resulting in cost savings and reduced environmental impact [23,47]. The effectiveness of nanoparticles as fuel additives can depend on various factors such as nanoparticle type, concentration, dispersion quality, and compatibility with existing fuel infrastructure and engine technologies. Additionally, potential challenges such as nanoparticle aggregation, compatibility issues, and environmental concerns must be carefully addressed in developing and deploying nanoparticle-enhanced fuels.

## 4 Conclusion

The photocatalytic efficiency of rod-shaped bimetallic NiMn_2_O_4_ NPs in the degradation of AG 25 dye was assessed, which was synthesized using a hydrothermal method. Under optimum levels of H_2_O_2_ concentration (0.3 M) and catalyst dose (0.05 mg/mL), the NPs showed a maximum removal efficiency of 96 % in 40 min at 315 K. AG-25 dye degradation followed pseudo-first-order kinetics. The enhanced light absorption in the NiMn_2_O_4_ NPs is ascribed to the efficient separation of charge carriers in the NiMn_2_O_4_ NPs, which leads to superior photocatalytic performance. Thermal catalytic degradation is an entropy-driven, spontaneous process that is enhanced at higher temperatures. NiMn_2_O_4_ NPs showed a promising impact on fuel properties as an additive, which increased fuel efficiency. As the concentration of NiMn_2_O_4_ increased, the fuel's flash and fire points decreased, and its pour and cloud points also showed a declining trend. The fuel's kinematic viscosity, specific gravity, and calorific values enhanced as the concentration of NiMn_2_O_4_ NPs was increased. NiMn_2_O_4_ NPs thus exhibit potential as effective photocatalysts for wastewater pollutant removal as well as fuel additives.

## Data availability

Data included in article/supp. material/referenced in the article.

## CRediT authorship contribution statement

**Shumaila Fatima:** Writing – original draft, Investigation. **Mahwish Iqbal:** Formal analysis, Data curation. **Haq Nawaz Bhatti:** Supervision. **Norah Alwadai:** Software, Methodology, Writing – review & editing. **Maryam Al Huwayz:** Validation, Formal analysis, Data curation, Funding Acquisition. **Arif Nazir:** Writing – review & editing, Visualization, Validation, Formal analysis. **Munawar Iqbal:** Writing – review & editing, Visualization, Validation, Formal analysis.

## Declaration of competing interest

The authors declare that they have no known competing financial interests or personal relationships that could have appeared to influence the work reported in this paper.
